# Resuscitation policy should focus on the patient, not the decision

**DOI:** 10.1136/bmj.j813

**Published:** 2017-02-28

**Authors:** Zoë Fritz, Anne-Marie Slowther, Gavin D Perkins

**Affiliations:** 1 Warwick Medical School, Division of Health Sciences, Gibbet Hill Campus, Coventry CV4 7AL, UK; 2 Cambridge University Hospitals; 3 Heart of England NHS Foundation Trust, University of Warwick

## Abstract

**Zoë Fritz and colleagues** discuss new approaches to resuscitation decisions that incorporate broader goals of care

Do not attempt cardiopulmonary resuscitation (DNACPR) decisions are made commonly in healthcare but can be a source of ethical concern and legal challenge. They differ from other healthcare decisions because they are made in anticipation of a future event and concern withholding, rather than giving, a treatment. DNACPR decisions were introduced to protect patients from invasive treatments that had little or no chance of success. However, inconsistencies in decision making, communication, and documentation have led to misunderstandings about what DNACPR means and to delivery of poorer care to some patients. Here we discuss the problems with current practice and outline newer approaches that place the patient, and their family, at the centre of the discussions. We focus on overall treatment plans and supporting clinicians and patients to make shared decisions about emergency treatments.

## DNACPR decisions

CPR is an invasive medical treatment that was never intended to be given to patients who are dying from an irreversible condition.^1^ DNACPR decisions provide a way of communicating when patients should not receive CPR, either because they do not want it or because it has little chance of success (box 1). They are an important mechanism for protecting patients from harm, but they have taken on practical, legal, and emotional significance far beyond their intended remit.^6^

**Box 1: Clinical context of CPR and DNACPR**

DNACPR decisions are considered in three situations:
  when a patient with capacity refuses CPR or a patient without capacity has recorded their refusal of CPR in advance
  when CPR is judged very unlikely to be effective because the patient is dying from an irreversible condition
  when the potential burdens of CPR outweigh the potential benefits
DNACPR policies are in widespread use. They exist in many countries,^2^ and 80-90% of those who die in hospital have a DNACPR in place^3^
a primary focus on acute care settings and a lack of consistency in policies between care settings is still widespread
One in five CPR attempts made in hospital result in survival.^4^ Average survival rate in the community is one in 10^23^
The decision not to attempt CPR should be distinct from decisions to initiate palliative care or to withhold other treatments
Many patients with DNACPR decisions are discharged from hospital^5^
Standardised DNACPR forms (fig 1) are often used to provide immediate access to decisions in the event of a cardiorespiratory arrest

**Figure f1:**
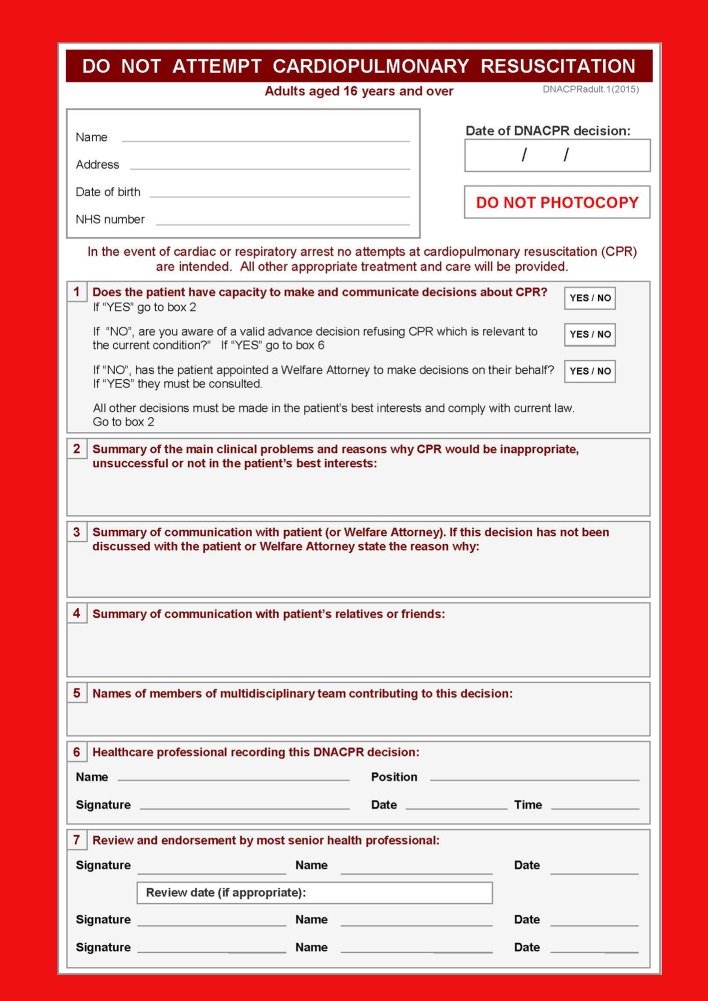
**Fig 1** DNACPR decision form

A comprehensive review in the NHS found shortcomings in considering, discussing, and implementing DNACPR decisions, as well as unintended consequences.^7^ The effects on patients and clinicians can be divided into three broad domains: futile or inappropriate CPR attempts, difficult and delayed discussion around DNACPR decisions, and inappropriate withholding of other treatments.

Firstly, we know that frailty^8^ and comorbidities^9^ are associated with worsening outcomes after cardiac arrest, and receiving attempted CPR when it has little prospect of success is one of the major concerns expressed both by patients approaching the end of their natural lives and by their relatives.^7^ This concern is well founded, as such attempts continue to take place.^10^ The ethics of widespread and indiscriminate use of CPR without balancing benefit with harms has been challenged.^11^

Secondly, doctors are often hesitant to initiate conversations about DNACPR owing to concerns about causing distress to the patient or fear of complaints.^2^ ^7^ Patients rarely initiate conversations, even though research shows that they would like to discuss CPR.^12^ Changing the focus of discussion from specific treatment options to acceptable health states and valued life might be more acceptable to them.^13^ Some doctors don’t understand the legal position of patients and families in making DNACPR decisions. The legal requirement to involve patients in DNACPR decisions varies across jurisdictions. In some countries DNACPR is prohibited,^2^ in others patients must consent to a DNACPR decision. Doctors in the UK must consult the patient or their family when writing a DNACPR notice unless doing so would cause “physical or psychological harm.”^14^

Finally, doctors and nurses can sometimes conflate DNACPR decisions with end-of-life care and mistakenly think that other treatment should not be given. Scenario studies show that presence of a DNACPR note makes doctors significantly less likely to take blood cultures, put in a central line, or refer to an intensive care unit.^15^ Nurses were significantly less likely to perform a variety of monitoring tasks and interventions for patients with a DNACPR decision than for those without.^16^ In clinical practice, patients with heart failure and a DNACPR decision were less likely to have their left ventricular function assessed or to receive secondary prevention than matched counterparts without such notices.^17^ A study of referrals to a medical intensive care unit showed that a DNACPR notice was the only factor significantly associated with a decision to refuse a patient admission.^18^

Clinicians are presented with an ethical dilemma: if they do not discuss CPR with a patient and record a decision, the patient may receive CPR that doesn’t work or that results in a quality of life that may not be acceptable to them; if they do, others may misinterpret it and compromise the patient’s overall care. Deciding what would achieve overall benefit for each person is compounded by the uncertainty of predicting future events. DNACPR practice as it currently stands raises considerable ethical concerns. Shifting the focus from a specific decision about CPR to making personalised plans on broader emergency care and treatment will help to tackle some of these concerns.

## Integration with advance care planning

DNACPR decisions have historically been separate from advance care plans. Some primary care doctors have been encouraged or incentivised to consider both for certain patient populations.^19^ Synergy with advance care planning can be achieved by putting CPR decisions in the context of overall goals of care and combining them with discussions about what treatments (or outcomes) a patient would or would not want. This kind of holistic approach has been adopted in Canada (box 2) and in paediatrics (box 3).

**Box 2: Alternative approaches in North America**

The Physician Order for Life Sustaining Treatment (POLST; http://polst.org/about-the-national-polst-paradigm/what-is-polst/) was developed in Oregon with the aim of ensuring that patient preferences for end-of-life care (including whether they wanted CPR) were honoured. It is intended for those who are seriously ill or frail. POLST, or versions of it, are now established in more than 20 US states.

In Canada, an adapted version—Medical Orders for Scope of Treatment (MOST; https://www.interiorhealth.ca/YourCare/PalliativeCare/ToughDecisions/Documents/MOST%20ACP%20cycle%20for%20patients.pdf)—is used to encourage people to “talk early, talk often.” The MOST form has specific tick boxes for treatments such as blood products and dialysis. It is supported by excellent infographics showing how advance care planning, MOST, and goals of care can be integrated.

**Box 3: Babies, children, and young people**

Advance care planning is well established in babies, children, and young people; in many areas it is delivered using standardised documentation; eg, the Child and Young Persons Advance Care Plan (CYPACP: http://cypacp.nhs.uk), Deciding Right (http://www.nescn.nhs.uk/common-themes/deciding-right/, or, in Scotland, Child and Young Persons Acute Deterioration Management CYPADM (http://www.gov.scot/Topics/Health/Quality-Improvement-Performance/peolc/CYPADM)
A complex interplay exists between the decision making roles of the child and their parents, especially where older children are involved
Widespread practice includes a third resuscitation decision that specifies some, but not all, resuscitative measures as appropriate. This is specifically captured in CYPACP paperwork
Legal status of 16 and 17 year olds varies considerably by country: it is important to be familiar with local legislation in this area
Courts are increasingly being used to resolve conflicting views regarding appropriate life sustaining therapies

## Alternative approaches

Several alternative models to DNACPR have been developed (boxes 2 and 4). On the POLST form, doctors are asked to record whether to attempt CPR and to tick a box to clarify whether patients would want comfort measures, limited treatment, or full treatment and whether they would want artificially administered nutrition. A systematic review of 23 research studies found that POLST is used most frequently by elderly, white patients approaching the end of their lives.^21^ Conversations about POLST are usually initiated by a non-physician facilitator who prepares the form for review and sign off by a clinician. The forms allow around 35 combinations of treatment preferences to be recorded. Do not attempt resuscitation, comfort measures, and withholding of antibiotics and artificial nutrition are recorded by approximately one third of patients. Many patients who made the decision to withhold CPR have said they would like to receive full active treatment in one or more sections of the form, emphasising the need to include recommendations about other aspects of care along with resuscitation guidance. The review found that POLST is generally well received by clinicians; they have confidence that it reliably records patient preferences and helps inform the delivery of overall care and treatment. Concerns included managing family disagreements, inadequate education for providers, and the time taken to complete POLST. Although POLST seems to be effective at guiding treatments that are consistent with the documented “orders,” more research is needed to examine the quality of decisions and to explore patient and family members’ experiences.

**Box 4: Alternative approaches in the UK**

All of these approaches replace isolated resuscitation decisions with broader goals of care, encourage earlier conversations with patients, and facilitate clear handover.

*Universal Form of Treatment Options (UFTO)*—UFTO was developed in Cambridge University Hospitals from focus groups with clinicians and patients and was informed by behavioural economics literature.^20^ UFTO is for all patients in a hospital setting, not just those approaching the end of life. The form provides a dichotomous choice between goals of care (active treatment or optimal supportive care), a box in which more specific or nuanced instructions can be written, and documentation of the CPR decision
*Treatment Escalation Plans (TEPs)*—TEPs were introduced as a replacement for the DNACPR process at Torbay Hospital, South Devon, in 2006. Their use spread locally in 2012 to cover all health providers in the acute and community sectors across Devon (population 1.1 million). Many local care homes have embraced the concept; 30% of elderly inpatients now arrive at Torbay Hospital with a TEP.
*Unwell and Potentially Deteriorating Patient Plan (UP)*—UP was developed in oncology at Gloucestershire Hospitals NHS Trust and has been further refined through a multidisciplinary working group including representatives from intensive care, palliative care, medicine, and surgery. UP includes explicit guidance on escalation of treatment. Evaluation of and feedback on UP have been positive; rates of CPR discontinued on grounds of the National Cardiac Arrest Audit criterion of “futility” have fallen from 17% in 2011 to 2% in 2016
*Deciding Right*—In the north east of England, Deciding Right puts CPR decisions into a wider context of planning emergency care in advance and in the context of mental capacity legislation. A free app is available to aid decision making. See http://www.nescn.nhs.uk/common-themes/deciding-right/

The Universal Form of Treatment Options (UFTO) was developed iteratively for hospitals through a modified Delphi approach with doctors, nurses, and patients. It sets the overall goals of care as “active treatment” or “optimal supportive care” and is considered for all patients who are admitted to hospital with an acute illness. A mixed methods evaluation reported that 82% of patients had UFTOs, a quarter of whom recorded decisions to withhold CPR.^22^ Frequency and severity of harms experienced by patients were significantly reduced when the recommendation not to attempt CPR was recorded within overall goals of care on an UFTO rather than on a standalone DNACPR. Interviews with clinicians and observation of ward practice showed that the UFTO helped provide clarity of goals of care and reduced negative associations with resuscitation decisions for clinicians. It changed the subject of conversations at nurse handover from resuscitation decisions to the patient’s condition and overall goals of care. This qualitative work provides a suggested mechanism for the observed reduction in harms.

## Towards a solution: development of ReSPECT

Patients, clinicians, healthcare commissioners, and regulators came together in a 100 strong meeting in 2014 to consider the role of DNACPR decisions, following a review of published evidence and evaluation of their use in the NHS.^7^ The group agreed that patient and family involvement in decisions needed improving and that resuscitation decisions should be considered in the context of overall treatment plans.

After this meeting 37 stakeholders (including patient advocates) convened regularly to develop an approach that could meet the needs of different care settings and travel with the patient. They used the approaches above as the starting point and drew upon examples of best practice in the UK and internationally. They agreed that, although a standardised framework is required, the approach should focus on individual preferences and tailored clinical guidance rather than standardised forms, encouraging person centred planning and care.

The group agreed that the aims should be to contextualise resuscitation decisions among overall goals of care; facilitate early discussion with patients and their families; and restrict documentation to a single sheet of paper (or digital equivalent), for access in an emergency. A public consultation process attracted over 1000 responses. The vast majority (91%) of respondents agreed with the aims. Inclusion of the terms “recommended” (to explain that the plan is not legally binding) and “summary” (to emphasise that more detailed information should be recorded in health records and in advance care plans) led to the acronym: Recommended Summary Plan for Emergency Care and Treatment (ReSPECT; www.respectprocess.org) (fig 2).

**Figure f2:**
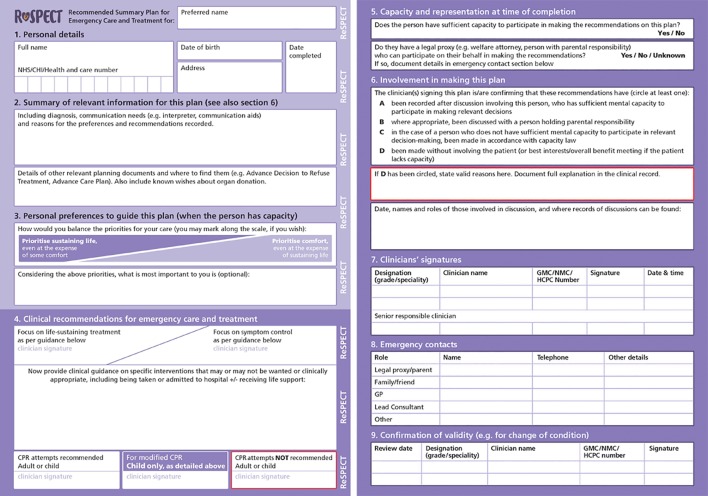
**Fig 2** ReSPECT form

ReSPECT was designed not only to replace DNACPR forms but to provide additional support for conversations about goals of care and to provide guidance to clinicians about which treatments would or would not be wanted in an emergency in the event of a patient not having capacity to make decisions for themselves (box 5).

**Box 5: ReSPECT development**

The group aimed to establish an approach which:

is developed with and is acceptable to patients, those important to them, healthcare professionals, carers, and other members of the public
includes a decision support framework that supports patients and clinicians to have informed discussions about benefits and burdens of emergency treatments including CPR
ensures that dialogue between the patient and clinicians is central to decision making
can be used across all care settings
can be used for people of all ages
is based on evidence and experience from other successful initiatives
contextualises a decision about CPR within overall goals of care, focusing on choices of treatments to be given rather than specifically on withholding CPR
Records patient preferences and treatment decisions to guide clinicians in an emergency when the patient lacks capacity to make decisions for themselves

The ReSPECT process can be initiated in different care settings, including admission to hospital, in the community or outpatient clinic for patients with chronic or life limiting conditions, or at admission to a care home. When these discussions are initiated with people who are well there is a risk that they will underestimate the state of ill health that they will tolerate and how many interventions they might want.^23^ ^24^ However there may be benefits of thinking through possible future illnesses; the earlier conversation may prepare the person for the acute situation. Ideally the conversation should begin early with a known clinician and should be revisited when there is a change in situation—for example, admission to hospital. We have provided guidance on discussing resuscitation and other treatment decisions elsewhere, including more detail about the ReSPECT process.^25^

ReSPECT tackles some of the barriers to having meaningful conversations about resuscitation and other treatment decisions, but logistical and ethical challenges remain. Community services are under pressure, so finding time to have adequate conversations may be difficult. Robust evaluation of the effectiveness of ReSPECT in achieving its overall goals will be essential.^19^

## Time for a change?

Given the weight of evidence against DNACPR decisions being made in isolation, how much (and what kind of) evidence is needed before a new approach is adopted? Some of the principles underpinning the new approaches to resuscitation decisions are already widely accepted—clinicians need to understand what is important to each individual patient and to advise their patients which outcomes are clinically possible or likely. Others are drawn from the research literature—conversations should be undertaken proactively before a crisis occurs; the option of attempting CPR should be discussed with more people, not just those needing DNACPR decisions or approaching the end of life; resuscitation decisions should be contextualised within overall goals of care. The aim of ensuring that recommendations are documented in such a way that patients receive the right treatments at the right time is one which is universally accepted. Changing the culture of resuscitation decision making will not be easy, but newer approaches may offer a step towards achieving this.

## Notes

Contributors and sources: ZF has conducted work on the ethical and practical issues around DNACPR orders and co-developed and evaluated the UFTO, as well as being in the working group for the development of ReSPECT. She has worked with GDP and A-MS on the NIHR evidence synthesis of DNACPR order use. GDP led the NIHR funded evaluation of DNACPR decisions in the NHS and was a member of the ReSPECT working group. A-MS was a co-investigator and ethics lead on the NIHR funded evaluation of DNACPR decisions in the NHS. ZF, A-MS, and GP discussed the content and structure of the article based on evidence from their previous work. David Pitcher, co-chair of the ReSPECT working group and retired consultant cardiologist reviewed and advised on wording of the manuscript; ZF wrote the first draft; ZF, A-MS, and GDP all contributed to further drafts.

Patient involvement: Members of the public and patients’ representatives were involved in the analysis of the problems with DNACPR orders, the development of UFTO and the development of ReSPECT. This manuscript was reviewed and approved by one of our patient collaborators.

Provenance and peer review: Not commissioned; externally peer reviewed.
